# Predictive Modeling of West Nile Virus Transmission Risk in the Mediterranean Basin: How Far from Landing?

**DOI:** 10.3390/ijerph110100067

**Published:** 2013-12-20

**Authors:** Véronique Chevalier, Annelise Tran, Benoit Durand

**Affiliations:** 1 Cirad, UPR AGIRs, Montpellier F-34398, France; 2 Cirad, UMR TETIS, Montpellier F-34398, France; E-Mail: atran@cirad.fr; 3 Anses, Epidemiology Unit, Laboratoire de Santé Animale, Université Paris-Est, Maisons-Alfort F-94706, France; E-Mail: Benoit.DURAND@anses.fr

**Keywords:** West Nile, Mediterranean basin, transmission, landscape, risk, model, prediction

## Abstract

The impact on human and horse health of West Nile fever (WNF) recently and dramatically increased in Europe and neighboring countries. Involving several mosquito and wild bird species, WNF epidemiology is complex. Despite the implementation of surveillance systems in several countries of concern, and due to a lack of knowledge, outbreak occurrence remains unpredictable. Statistical models may help identifying transmission risk factors. When spatialized, they provide tools to identify areas that are suitable for West Nile virus transmission. Mathematical models may be used to improve our understanding of epidemiological process involved, to evaluate the impact of environmental changes or test the efficiency of control measures. We propose a systematic literature review of publications aiming at modeling the processes involved in WNF transmission in the Mediterranean Basin. The relevance of the corresponding models as predictive tools for risk mapping, early warning and for the design of surveillance systems in a changing environment is analyzed.

## 1. Introduction

West Nile fever (WNF) is an arbovirosis caused by the West Nile virus (WNV) (*Flavivirus*, *Flaviviridae*). The transmission cycle involves wild and domestic birds as main hosts and mosquitoes, mainly of the *Culex* genus, as vectors. Under favorable environmental conditions, this cycle may be amplified and lead to human and horse infections. The latter two are considered to be dead-end hosts [1]. Most human cases remain asymptomatic. However, around 30% of infected people get sick, with symptoms ranging from a flu syndrome to encephalitic diseases, with recent reported case fatality rates ranging from 3 to 17% [2,3]. Ten percent of horses infected by WNV present neurological disorders [4,5]. Consequently WNF is a veterinary and human public-health issue.

The phylodynamics [6] of WNV has been analyzed in several studies, according to which WNV originates from sub-Saharan Africa where it circulates in an endemic cycle [7] . Introductions of WNV from this cradle occurred regularly in Western Europe (via Maghreb) and in Eastern Europe (via Middle East). Two major lineages are distinguished: lineage 1 (strains from all over the World) and lineage 2 (strains from Africa and Europe) [8]. Lineage 1 further divides into 3 subclades: subclade 1a (the main branch of the lineage 1) [9], subclade 1b (Kunjin strains from Australia) and subclade 1c (strains from India).

In 1999 a WNV strain belonging to lineage 1a was introduced into north America and spread throughout the continent to reach the western coast 3 years later. Between 1999 and 2010, around 1.8 million people were infected, with more than 12,000 encephalitis/meningitis syndromes and 1,308 deaths [10]. This 10-years WNV circulation period was characterized by a high variability in the intensity of local transmission on several spatial scales and between years, and by waves of wild bird mortality [11].

WNV has been circulating in the Mediterranean Basin at least since the 1960s. Most of human and/or equine cases were caused by strains belonging to lineage 1a, characterized by a moderate pathogenicity for horses and humans and a limited or no pathogenicity for birds [12]. However, since 2000, and in particular since 2010, WNV epidemiological pattern has evolved from a very low level of endemicity without any bird mortality to a sudden increase of this mortality and a higher incidence of animal and human neurological cases. Furthermore, lineage 2 strains, so far confined to the south of the Sahara, have been recently detected in central Europe (Hungary [13], Austria [14], Greece [15], Italy [16]). The major WNF epidemic that occurred in 2010 in Central Macedonia, Greece, was caused by a lineage 2 strain [17,18]. Recently, human cases were reported from Albania, Hungary, Israel, Italy, Macedonia, the Palestinian territory, Romania, the Russian federation, Serbia, Spain, Ukraine, Tunisia, Turkey and Greece [12,19,20,21]. Most of the European countries have implemented surveillance networks, either passive of active, that have improved the quality of available epidemiological data. However outbreaks appear temporally and spatially unpredictable.

Statistical and mathematical models may allow predicting WNF occurrence risk and, more generally, the risk of WNV transmission between hosts (birds and incidental hosts) and vectors. Three modelling approaches are used by epidemiologists to predict the risk of case occurrence for a given disease, or the transmission risk of its agent: risk factor analysis, landscape epidemiology, and transmission dynamic modelling. Risk factor studies are observational studies performed in the natural environment of hosts: disease occurrence figures and data describing potentially linked factors are collected in naturally affected populations, and statistical models are used to explore the link between disease occurrence and covariates [22]. These models may be spatial or not, depending on the nature of the covariates. A complementary approach, so-called landscape epidemiology, takes into account spatial relationships between the components of the epidemiological system. The term of “landscape epidemiology”, attributed to the Russian parasitologist Pavlovsky [23], corresponds to the analysis of the geographic distribution of diseases as the result of several elements: the co-occurrence, at the same place, of animal donors, of vectors, of animal recipients and of the pathogenic agent itself; and the influence of local environmental conditions on the transmission of infection. The objective of landscape epidemiology studies, also called spatial epidemiology [24,25] is to produce risk maps for disease occurrence, or for the transmission of the disease agent. Mathematical models of disease transmission explicitly represent the health states of the individuals of the population in which the disease agent circulates. Most of the time, the health state—susceptible, infectious, removed/recovered, are discrete and are also called “compartments”, hence the name of “compartmental models” for most epidemiological models. Transitions between health states, that describe the transmission processes and the natural history of the disease, are often represented by differential or by difference equations; the parameters used to model these transitions as well as the host and vector population dynamics are measured in field or laboratory studies or are obtained from experts’ opinion. Mathematical models may incorporate the whole epidemiological system or a specific part of it. They may address the circulation of the disease agent in a single population, in several interacting populations (meta-population models) that may be spatially defined (spatialized models). Whatever the case, these models may be used to test some transmission scenarios, to predict the disease incidence in various environmental contexts, or to assess the impact of intervention strategies [26]. Here we focus our analysis on the specific case of WNV in Europe. A more extensive review of mathematical models of mosquito-borne pathogen transmission can be found in [27].

In this paper we propose a systematic review and analysis of the published studies belonging to each of the 3 previously mentioned approaches, applied to countries or areas of the Mediterranean basin, and dedicated to the risk of WNF occurrence or of WNV transmission. The relevance of the corresponding models as predictive tools for risk mapping, early warning and for the design of surveillance systems is analyzed.

## 2. Method

PubMed and ISI Web of Knowledge were searched, from 2000 till September 2013, using the terms “West Nile” and, separately “model”, or “spatial”, or “risk factors” or “Europe”, using the “all fields” option to allow retrieval of articles in which the search terms appeared in the titles, abstracts, or keywords. Abstracts retrieved were read by the two same persons, and inclusion and exclusion criteria were applied to identify the final list of publications for full text reading. Inclusion criteria were articles using statistical and mathematical modelling approaches to model WNF risk in animals or humans. Reviews, statistical analysis and models built in a north American context, outbreak notifications, prevalence studies, descriptions of clinical disease, pathogenicity and diagnosis in humans or animals, experimental infections in animals, development of vaccines, genome sequencing alone, entomological surveys alone were discarded. The relevant papers cited by selected articles, but not identified by the above selection procedure were included in the review. Only English written articles were included. 

## 3. Results

The first request, “WN AND models”, allowed to identify 32 papers, among which 27 were theoretical studies, built with US data or studied the risk of introduction of the virus into Europe. The second request, “WN AND Europe”, identified 24 papers: 13 were deleted because they were theoretical, descriptive or phylogenetic studies. Thirteen papers were identified through the “WN and risk factors” request. Eleven were discarded for the same reasons. The last request, “WN and Europe”, selected 5 papers from which 3 were deleted. Once duplicates had been deleted, and new relevant papers cited by selected articles added, 18 papers were actually examined. Eight were risk factor studies performed in France, Italy, Spain, Tunisia, Morocco and Iran (Table 1), 5 landscape epidemiology studies performed with French, Italian, Spanish or Israeli data (Table 2) and 5 concerned mathematical models, the estimation of their parameters and their use (Table 3).

### 3.1. Risk Factor Analyses of WNF Occurrence and/or Transmission

Although recently changed, the epidemiology of WNV in the Mediterranean Basin is very different from that prevailing in north America. Bird mortality and infection rates in mosquitoes are high in north America [28], and the WNV infection rate in mosquito vectors is thus commonly used as an indicator of WNV transmission intensity [29]. Similarly, the mortality fraction in birds can also be used as an indirect indicator of WNV transmission, as in Southern Ontario for the period 2002–2005 or in south Carolina in 2003 [29,30]. Oppositely, in the Mediterranean basin, these parameters are generally null or very low [31,32,33,34], with the recent exception of Hungary where neuroinvasive WNF was diagnosed all over the country in dead goshawks and other birds of prey, the estimated infection rate in mosquitoes being 5% [35]. Therefore, in most countries of the Mediterranean basin, bird mortality or mosquito infection rate cannot be used as a proxy for WNV transmission, and case occurrence data (seroprevalence or clinical cases) in incidental hosts (horses or human) are used instead. It was the case for the 8 risk factors studies reported here, of which 6 used horse seroprevalence data as a proxy for WNV transmission risk, whereas 2 used data about human and/or horse clinical cases (Table 1). Three categories of potential explanatory variables were analyzed in these 8 studies: abiotic parameters (such as rainfall and temperature), land cover characteristics (such as vegetation type, presence of water bodies), and landscape indices (such as landscape fragmentation indices). In several studies, variables describing differences in host exposure (such as, for horses, the breed, the age, or the housing conditions) were also included to adjust the effect of abiotic, land cover and landscape variables.

**Table 1:** List of articles aiming at identifying the risk factors of WNF occurrence and/or transmission, and variables associated with seroprevalence or case occurrence in human or horses. Ref. stands for References.

Infection Marker	Scale	Explicative Variables				Validation	Prediction	Ref.
		Abiotic	Landcover	Landscape	Other			
Horse seroprevalence	Local (France)				Age, breed, group size	Yes, internal	No	[36]
Horse cases and seroprevalence	Local (France)		Wet sansouire, open water, rice fields, dry bushes			No	No	[37]
Horse seroprevalence	Local (France)		Density of hetero-geneous agricultural areas	Insterspersion and juxtaposition index		Yes, internal	Yes	[38]
Horse seroprevalence	Local (Iran)	Elevation			Age	No	No	[39]
Horse seroprevalence	Local (Spain)				Number of horses within the holding, transport within the last 6 months, presence of mosquitoes	No	No	[40]
Horse seroprevalence	Country (Tunisia)		Night-time land surface temperature, biannual phase of NDVI		Distance to the nearest RAMSAR site	Yes, external	Yes	[41]
Horse cases	Local (Morocco)		NDVI, rainfall			No	No	[42]
Human and horse cases	Continental (Russia, Greece, Israel, Romania, Turkey, Hungary, Italy, Spain)	Temperature, Relative Humidity				No	No	[43]

**Table 2:** List of articles aiming at producing risk maps for the transmission of WNV or WNF disease. Ref. stands for References.

Scale	Wild Birds		Mosquitoes		Risk Indices/Model	Ref.
	Species	Abundance Model	Species	Abundance Model		
Local (France)	60 species	Qualitative probability of presence according to land cover (6 classes)	*Cx. pipiens* *Cx. modestus*	Qualitative density level (5 classes), data: bird-baited trapping	Vector and host occurrence probability indexes, host richness and abundance indexes	[44]
Country (Israel)			*Cx. pipiens*	Spearman and Pearson correlation with temperature and precipitation		[45]
Local (Italy)			*Oc Caspius* *Cx. pipiens* *Cx. modestus*	Bayesian Generalized Linear Mixed Model (GLMM) of CO_2_-baited trapping data according to elevation, rainfall, temperature, NDVI, season		[46]
Local (Italy)			*Cx. pipiens*	GLMM		[47]
Local (Spain)	32 migratory species, present in large numbers, associated with aquatic habitat	Presence/absence: only abundant species (>2,000 pairs) are addressed	*Cx. pipiens*	Weighted Linear Combination (WLC) of temperature, rainfall rate, distance to the nearest humid area	WLC of wild bird presence, *Cx. pipiens* abundance and equid density	[48]

**Table 3:** List of articles addressing the respective roles of vector species and of wild bird species in WNV transmission, and WNV transmission dynamics. Ref. stands for References.

Study Type	Method			Ref.
	Species/Genus	Explanatory/Calibrated Variables	Method	
Respective roles of wild bird species	25 Bird species	Migrating status	*t*-test	[49]
	72 Bird species	Migrating status Body weight	GLMM	[50]
Respective roles of mosquito species	Duck, Horse *Cx. pipiens*, *Cx. modestus*, *Ae Caspius*, *Ae. vexans*	Host abundance and biomass	Multi-host model of host choice by vectors	[51]
WNV transmission dynamics	Passerines *Cx. pipiens*/*Cx univittatus* Horses Chicken	Vector-host ratio in each population Nestling-adult bite relative risk Passerine-incidental host bite relative risk	Meta-population model (3 vector populations, 5 host populations)	[52]
			Use of a meta-population model	[53]

The influence of abiotic parameters was analyzed in 4 surveys, of which 1 was conducted at the continental scale [43], and 3 covered specific countries: Morocco [42], Tunisia [41] and Iran [39]: Paz *et al.* analysed a dataset of laboratory-confirmed human and equine cases from Russia, Greece, Israel, Romania, Turkey, Hungary, Italy and Spain reported during summer 2010, with regards to spring and summer temperatures, relative humidity and rainfall. Data were analyzed using Pearson and lag correlation as well as multinomial logistic regressions. A positive correlation was observed between WNF cases and temperature. Northern countries displayed strong correlations with a lag of up to 4 weeks whereas cases occurred immediately after temperature anomalies in southern countries. The association between WNF cases and relative humidity (RH) was weak. Rainfall seemed to have had no impact on human WNF occurrence. According to the existing surveillance systems, WNF cases appeared 3 weeks later in horses than in humans, without any association with temperature or RH [43]. Temperature conditions prevailing in a given area may be measured using an index such as the night-time Land Surface Temperature (LST), computed from remote sensing imagery. This index was used in the Moroccan and Tunisian studies because the main activity period of mosquitoes ranges from sunset to dawn. In Morocco, night-time LST did not seem to have an influence on WNV transmission to horses, whereas the role of rainfall was unclear [42]. In Tunisia, the night time LST was positively associated with a higher prevalence rate in horses [41]. In Iran, the elevation (which is linked to temperature) was negatively associated with the seroprevalence in horses [39]. 

Temperature is known to influence mosquito population dynamics, increasing the reproduction rate, the number of blood meals, and the duration of the breeding season. Higher temperatures are also known to increase vector competence, thus the transmission intensity, by reducing the extrinsic incubation period [54,55,56,57,58,59,60]. As confirmed by the examined studies, the influence of rainfall on mosquito population dynamic (thus on WNV transmission) is more controversial and depends, among others, on the mosquito species considered. Surface water is necessary to the mosquito larval development. Heavy rainfall increase the surface of standing water, thus favour mosquitoes that uses permanent water sources such as *Cx. hortensis*. On the other hand, heavy rainfall may also dilute the nutrient for larvae thus decrease the development rate [61]. From an epidemiological point of view, drought was shown to favour the WNV transmission in the US, as standing water becomes richer in nutrients. On the other hand the reduction of standing water surfaces increase the bird concentration around these water holes, thus increase the contact between hosts and vectors [57,62,63].

The transmission of West Nile virus (WNV) depends on the co-occurrence in space and time of both susceptible avian reservoir hosts and competent mosquito vectors. Besides abiotic factors, both are influenced by geographic variables such as land use/land cover. Land cover may be characterized by indexes such as the normalized difference vegetation index (NDVI) that quantify the local photosynthetic activity, and can be computed from remote sensing images. In Morocco NDVI values were significantly higher in a 10 km radius around horse cases for 2003 and 2010, the years of WNF occurrence [42]. In Tunisia, higher values of the biannual phase of NDVI were associated with higher values of seroprevalence rate in horses, meaning that humid late spring and fall were more favourable to WNV transmission than drier occurrence of these seasons [41]. Both effects are related to vector population dynamics: high NDVI values indicate a higher photosynthetic activity, hence the presence of breeding and resting sites for mosquitoes; seasonal differences of NDVI values are known as predictive factors for mosquito abundance [64]. Besides indices such as NDVI, satellite imagery may be used to classify land cover into types, such as wetlands or agricultural areas, and the surface covered by each type may be measured. In Camargue (France) in 2004 the relationships between seroprevalence in horses and the surface locally covered by land cover types were analyzed using a generalized linear model. Rice fields and dry bushes, wet ‘sansouire’ and open water were shown to be the major components of the landscapes associated with high seroprevalence [37]. It is well known that potential mosquito vectors oviposit in specific habitats. For instance, *Aedes* larval habitats are constituted by brackish temporary ponds and low “sansouire” in Camargue whereas *Cx. modestus* species prefer quiet permanent wet areas such as rice fields, reeds or ponds containing low level salt. On the other hand, many wild bird species are attracted by wetlands that constitute adapted breeding sites [37]. 

Landscape metrics describe and quantify the spatial configuration of landscape elements. Many landscape metrics have been defined to characterize the composition (nature and area covered by the various land cover classes) and the complexity (number, area and spatial interweaving of patches) of landscapes [65]. In France, and using a logistic model, the link between horse seroprevalence and landscape metrics was studied in 2 areas where horse cases had been detected (Camargue and the Var). Environmental data were derived from the CORINE Land Cover 2000 (CLC2000) database, provided by the European Environment Agency (EEA, 2007). A positive association between seroprevalence in horses and landscape complexity was observed [38]. After evaluation and internal validation, the model was used to build a prediction map for the areas at risk of endemic circulation of WNV along the Mediterranean coast. Other studies have shown that landscape metrics provide a useful quantification of variations in biodiversity or species richness. In particular, bird species richness was shown to be linked to landscape heterogeneity [66]. Similarly, landscape metrics have been linked to the abundance of Bluetongue vectors (*Culicoides imicola*) [67] and to the risk of Bluetongue clinical cases or seroprevalence [68,69].

Variables describing differences in host exposure were used in 4 studies dedicated to seroprevalence in horses. Beside spatial heterogeneity observed in the 4 studies, the location of the stable was taken into account in Tunisia: the distance to the closest Ramsar site (Ramsar International Convention on Wetlands of International Importance) was the most important explanatory variable [41]. The proximity to wetlands (favourable sites for mosquitoes) that present a higher abundance and diversity of birds thus increases the risk of WNV transmission [70]. A similar effect has been observed in France, 2003 [71]. The role of herd size was highlighted in France [36], and Spain [40], horses living in small herds being more at risk than those living in larger ones. Two main elements can explain this result: (i) horses living in larger herds benefit from better management practices and are thus less exposed to mosquito bites than small one, (ii) because of the low infection rate in mosquitoes, the individual probability of being bitten decreases when the herd size increases. Finally, age was statistically associated with seroprevalence in Iran [39], suggesting an intense and regular viral circulation, the risk of having experienced at least one infective mosquito bite increasing then with the age of the animals.

### 3.2. Landscape Epidemiology Studies

The development of Geographic Information System (GIS) software has greatly facilitated the representation and the treatment of spatial data with the superposition and the combination of layers, each dedicated to a specific element of the infection transmission process. For WNV, landscape epidemiology studies consider that the geographic variations of WNV transmission result from the variations of abundance in competent birds and vectors. The geographic distribution of the disease also depends on human and horse spatial density. Three types of layers are thus considered in WNV landscape epidemiology studies: the mosquito abundance, the wild bird abundance and the abundance of incidental hosts. Once separately mapped, these layers are superimposed and combined to compute and map risk indices.

In the published studies, the mosquito species considered in the vector abundance layer always included *Cx. pipiens*, the other mosquito species taken into account being *Cx. modestus*, *Oc caspius* and *Coquillettidia richiardii* (Table 2). Several approaches have been used to produce a map of the geographic variations of vector abundance. Tran *et al.* [44] based vector abundance maps on the classification of remotely sensed images into land cover classes. For each mosquito species (*Cx. pipiens* and *Cx. modestus*) and activity (breeding and host-seeking), a probability of occurrence (5 levels, from “never or accidentally collected” to “very high density”) was associated with each of the land cover classes, based on bird-baited trapping data and on expert knowledge. Spatial statistical tools were used to model the trapping data according to the local values of covariates [45,46,72]. Increasing elevation had a negative effect on vector abundance [46] whereas temperature (weekly mean [46,72] or positive anomalies [45]) and NDVI [46] were positively linked to vector abundance. Bisanzio *et al.* [46] also report a significant effect of the season (modelled as a sinusoidal curve having a 1 year period and peaking in the 1st week of August), suggesting that the other covariates they used did not capture the effect of season on abundance. Rainfall was positively linked to vector abundance in [46] (10 days cumulative rainfall), but negatively in [72] (4 weeks cumulative rainfall), no association being reported in [58]. In [46], these statistical associations did vary according to the vector species. Finally, other authors applied the landscape epidemiology approach to the abundance of vectors. Three layers were thus considered by Rodriguez-Prieto *et al.* [48], according to literature: distance to the nearest humid area, temperature and rainfall. They derived the vector abundance layer by computing a weighted sum of these layers (after having applied to each a normalization process), weights being based upon experts’ opinion.

The abundance of birds was taken into account in 2 studies. The number of considered bird species varied between 15 and 60 according to the study. Each of the studies used a specific approach for mapping bird abundance. Rodriguez-Prieto *et al.* [48] used maps from the Spanish national biodiversity inventory database to generate presence/absence maps for competent birds. As for vector abundance, Tran *et al.* [44] based bird abundance maps on the classification of remotely sensed images into land cover classes. For each bird species (n = 60), season and activity (feeding, roosting or breeding), a probability of occurrence (six levels, from “absent” to “very abundant”) was associated to each of the land cover classes, based on experts’ opinion. Because they address different geographic areas (Castille and Leon for the Spanish study, Camargue for the French study) and use different methods (data-based maps for the Spanish study, and maps based on ornithologists’ knowledge for the French study), both studies are difficult to compare. However, it is worth noting that none did use spatial statistical modeling techniques for generating the bird abundance map, a situation that differs from the case of vector abundance maps. Indeed, the use of space by wild bird species is certainly less directly influenced by climatic or vegetation variables than for mosquito species: this probably hinders the definition of statistical models of bird abundance based on environmental covariates [25].

Three of the studies considered in this section were dedicated to the identification of areas with high vector productivity, this productivity being considered a proxy for the risk of WNV introduction and amplification. Tran *et al.* [44] integrated the bird and vector layers, but did not propose any risk indicator. They rather used a descriptive approach, mapping season-specific vector and host occurrence probability indexes, as well as host species richness and abundance indexes. Rodriguez-Prieto *et al.* [48] explicitly took into account the density of horses to compute a WNF risk in horses. Three layers were thus considered in their study: wild birds, mosquitoes and horses. They proposed a risk computation procedure based on a weighted linear combination of these 3 layers (after normalization of each layer), the weights being based on literature and experts’ opinion. The corresponding maps showed high risk areas (such as Guadarrama and Cantabric mountains), with increased risk levels in July and August.

### 3.3. Integrative Studies

The foraging behavior of blood sucking arthropods is a major element that shapes the epidemiology of arboviruses. The intensity of WNV transmission at a given place and the risk for incidental hosts depends on the host preferences of the mosquitoes living there. It also depends on the availability of hosts: if the species spectrum of mosquitoes likely to transmit WNV is limited, it is not the case for wild birds: field studies allowed detecting anti-WNV antibodies in many of the nearly 1,000 bird species that live in Europe during all or part of the year. The intensity of WNV transmission at a given place thus depends on the local biodiversity of mosquitoes and of wild birds. Furthermore, it depends on the calendar date as the population dynamics of mosquitoes and wild birds is strongly seasonal in Europe. If no study has yet addressed these 3 elements simultaneously (biodiversity of vectors, biodiversity of hosts, population and transmission dynamics), some have been dedicated to each of them. In this section we review the studies that have addressed the respective roles of mosquito species in WNV transmission, the respective roles of wild bird species in WNV transmission, and the mathematical models of transmission dynamics (Table 3).

#### 3.3.1. The Respective Roles of Mosquito Species in WNV Transmission

Several methods allow studying the foraging behavior of blood sucking arthropods, such as direct observation, baited traps or analysis of blood content in mosquito gut. Analysis of blood meal data in mosquito communities suggests that the mosquito foraging behavior primarily relies on the availability of hosts in a given landscape [73,74]. However, this does not preclude the existence of innate host preferences for specific mosquito species. Host feeding pattern may be defined as the distribution of meals taken on different vertebrate hosts. It depends on 3 factors: the innate tendency of mosquitoes to be attracted by specific host species (host preference), the local presence of individuals that may be bitten (host availability), and the defensive behavior of these. Balenghien *et al.* [51] have proposed a theoretical framework for representing mosquito host-feeding patterns and for analyzing data obtained using host-baited traps and blood meal analysis. This framework separates host preference from host abundance. It thus allows computing host feeding patterns for various host populations (host species assembly and relative abundance of host species). This framework has been applied to bird-baited trapping data obtained in Southern France (Camargue) for several competent WNV mosquito species or groups of species: *Cx. pipiens*, *Cx. modestus*, *Ae. caspius* and *Ae. vexans*. The model was used to analyze the relative importance of these mosquito species in the enzootic circulation of WNV (quantified by the probability for a mosquito to feed twice on a bird) and in the infection of incidental hosts (quantified by the probability for a mosquito to feed on a bird and on an incidental host). Results show that the relative importance of each vector species varies according to the composition of the host population and on the ratio of the bird biomass to that of incidental hosts: if the enzootic circulation is always carried by *Culex* species, the role of bridge vectors (between birds and incidental hosts) would also be carried by *Culex* species when bird biomass is low in comparison with that of incidental hosts, whereas it would rather be carried by *Aedes* species in the opposite situation. Interestingly, intermediate values of the biomass ratio induce a lower ability of each mosquito species to endorse the role of bridge species. Bird abundance shows large variations during a year, with complex patterns induced by nesting periods (of which the start and end dates vary according to the species), dispersion and migratory behaviors (that also vary according to the species). Moreover, local bird abundance may also be influenced by human behaviors (agricultural practices, hunting). These abundance variations should thus induce variations throughout the year of the identity of bridge vector species and of the infection risk for incidental hosts.

#### 3.3.2. The Respective Roles of Wild Bird Species in WNV Transmission

Reservoir competence, the ability of a species’ to act as reservoir for pathogens and transmit them before or without dying, is a function of several parameters: the host susceptibility (probability of a host becoming infected by infected vectors), the host infectivity (probability of a vector becoming infected when feeding on an infected host), the duration of infectiousness (number of days a host remains infectious), and the level of viraemia which allows or not the biting mosquito to get the virus from the host [75]. Ecologists and epidemiologists have recently begun to consider reservoir competence as resulting from the life history characteristics of species [75]. Life history theory suggests the existence of tradeoffs between self-maintenance such as immune defenses and other activities such as reproduction and growth: “fast-lived” species would invest minimally in adaptive immunity, contrary to “slow-lived” species. As a consequence, the potential to transmit a given pathogen would be higher for “fast-lived” species than for “slow-lived” ones [75]. Serological studies conducted in wild birds provide information on their exposure to infectious bites, either in Africa (for migrating birds) or in Europe (for resident species). A study performed in Spain during the 2004 pre-nuptial migration period showed that trans-Saharan migrants were more frequently seropositive than resident birds [49]. In a second study [50], carried out in Spain between January 2003 and February 2005, the statistical association between seropositivity and life history traits was analyzed. The association between migratory behavior was confirmed and refined, with a lower prevalence in winter migrants (birds coming from central and northern Europe) than in resident birds, and, oppositely, a higher prevalence in summer migrants (birds wintering in Africa) than in resident birds. Seroprevalence was also associated with bird weight. This could be explained by both a greater longevity of larger birds and by a stronger attractivity of vectors by larger birds, due to a higher CO_2_ production. Interestingly, no association between seroprevalence and taxonomic level was observed, suggesting that differences between species are more attributable to life history traits than to genetic factors. 

Besides species-level life history characteristics, individual traits may also play a role in WNV transmission. For example, nestling may play a specific role in WNV circulation, because of a greater exposure to mosquito bites (absence of feathers, immobility), or because their immature immunological system may favor a prolonged viraemia, or thanks to a direct transmission between nestlings and their parents. Studies based on bird seroprevalence data, conducted in Africa and in north America, have suggested such a particular role of nestling [76,77]. However, field studies based on mosquito trapping did not show a greater attractiveness of vectors by nestling [78,79], even if recently developed trapping devices (nest mosquito traps [80]) may lead to different results [81]. 

#### 3.3.3. Mathematical Models of Transmission Dynamics

From an epidemiological perspective, the successful transmission of an arbovirus at a given location mainly depends on the spatio-temporal co-occurrence of vectors and hosts. Some dynamical processes may however induce important variations of risk: the vector-host co-occurrence depends on the mobility of hosts (including migratory behaviors of wild birds), on the behavior of incidental hosts that determines their exposure to infectious bites, and on the vector and host population dynamics. Mechanistic dynamic models allow representing such dynamical phenomena and their impact on WNV circulation. A metapopulation model has been proposed by Durand *et al.* [52] for the circulation of WNV between Western Africa and Western Europe. Three geographic locations were considered: an African wet area, an African dry area and a European area. Each had its own pattern of vector abundance (only *Culex* species were considered) with a yearlong presence of vectors in the African wet area peaking during the rainy season), this presence being seasonal in the African dry area (rainy season) and in the European area (March-October). Five populations of passerines were considered: 3 resident populations (one per geographic area) and 2 migratory populations, which linked the 3 areas together (between the African wet area and the European area, and between the 2 African areas). Bird population dynamics was explicitly represented in the model (with population-specific nesting periods) as well as WNV transmission dynamics between birds (with different probabilities of being bitten in nestling and adults) and between birds and horses. The model was calibrated using field data collected in Africa and in Europe (bird seroprevalence data), and validated using independent data (bird seroprevalence data, infection rates in vectors, seroprevalence and incidence data in horses).

The limit cycle (*i.e.*, the yearly dynamic once the equilibrium has been reached) was analyzed to compare WNV surveillance systems in the European context [53]. Optimal sample sizes of equivalent surveillance designs were computed. Comparison of the corresponding yearly costs showed that, where possible, the passive surveillance of horses by specialized veterinarians was the most cost-effective system in the European context.

## 4. Discussion/Perspectives

**Figure 1:**
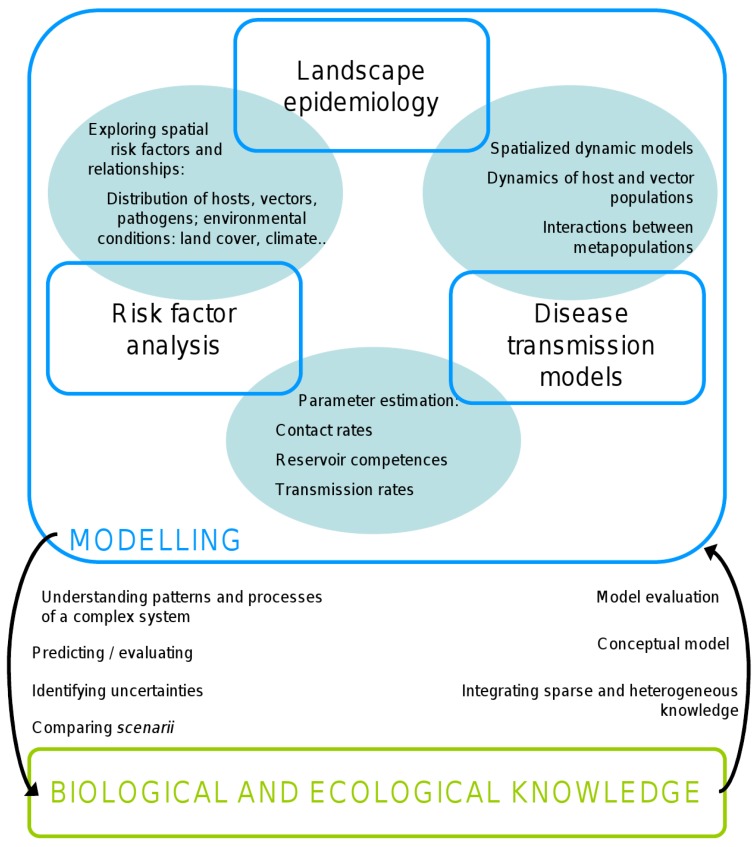
The complementary contributions of risk factor analysis, landscape epidemiology and disease transmission modeling to the biological and ecological knowledge of epidemiologic systems, and the mutual input of modeling and biology.

Notes: Papers of concern are listed in the corresponding tables: Table 1 for Risk factor analysis, Table 2 for Landscape epidemiology and Table 3 for Disease transmission models.

Even if the health impact of WNF is reduced in the Mediterranean Basin compared to what is observed in North America, the disease pattern changed during these past 20 years, with the first detection of the lineage 2, a sudden increase of the number of outbreaks and of neurological cases, either in human or horses, and an unexpected mortality in wild birds, in particular in Hungary. These changes raised concerns about the understanding and the prediction of outbreaks in order to implement adapted and cost-effective surveillance systems. In North America, many modelling studies have been conducted for the understanding, prediction and risk assessment of West Nile virus transmission and/or emergence. Models are either statistical (time/space) or process-based. Some are vector-based and aim at forecasting the dynamic of mosquito populations [82] or to spatially evaluate of the risk of acquiring infection from a mosquito bite [83]. Some are population-based and were elaborated to link WNF incidence with temperature data [84,85] or to simulate WNV circulation in an epidemiological system [86,87,88,89,90]. On the contrary, and probably because of the lower impact of WNV, relatively few studies have been performed in the context of the Mediterranean basin. Eighteen papers were examined, and classified in 3 groups, which contributions to the understanding of WNV transmission are highly complementary (Figure 1). 

The first examined group of studies aimed at investigating the potential association between biotic/abiotic factors and human/horses WNF cases or seroprevalence rates, using statistical methods. If not directly applicable to infer on geographical variations of the risk of WN transmission, the results of such studies help identifying the main factors involved, thus integrate these factors in predictive spatial and/or dynamic models. One of the most important results is the influence of temperature on the risk of WNF occurrence or of WNV transmission. As a matter of fact, and according to the World Meteorological Organization, warming trend in Europe and Eurasia is confirmed [43]. It is thus crucial to quantify the link between temperature and transmission risk, in order to build predictive models based on predicted meteorological data. The relevance of these results depends on the scale they were performed: if useful at the continental level [43], or at the national level [41,42], the relevance of this parameter at a local scale remains to be investigated.

Furthermore, the way the temperature variable is computed (mean weekly or monthly value, mean daily minimal or maximal) is important and the precise effect of each kind of index on vector biology needs to be further explored. Similarly to North America, the role of rainfall on WNV transmission in the Mediterranean Basin remains controversial. It is likely that rainfall influence depends, at least, on 3 factors: (i) the mosquito species considered since each mosquito species needs a specific biotope for larval development, and the soil composition which determine (ii) in what extent the water percolate or not thus lead to permanent water or not, and (iii) the organic composition of water. The study of Paz *et al.* is currently going on in the frame of Mobod project (Mosquito-Borne Diseases Determinants), granted by the European Centre for Disease Prevention and Control (ECDC). Once the link between climatic variables and outbreaks occurrence modeled, further step was to add in this model additional environmental variables. Besides the effects of temperature and rainfall, the results of the studies examining the impact of abiotic factors on WNV transmission were consistent with current knowledge on mosquito and wild bird ecology: the transmission intensity seems to be higher when located close to Ramsar sites (where bird biodiversity is higher), in biotopes favourable either to mosquito or wild bird presence (rice fields for instance or fragmented habitats), and where the value of NDVI index is increased.

From a public or animal health point of view, either human or horse cases occurrence or seroprevalence rates are the most exact surrogates of the risk of infection of horse and human populations. Results can be used for surveillance and control action provided that confusion variables are taken into account, such as host sensibility and exposure level. Horse vaccination is allowed in Europe since 2009. Vaccination coverage is unknown, but the use of vaccine largely varies across countries. Serological results obtained in horses after 2009 should thus be interpreted cautiously, taking into account previous vaccinations. Horse exposure mostly depends on their close environment and on the management practices. Regarding to humans, behaviour may strongly determine the exposure level, but many other features may modify the bite intensity, such as feeding behaviour of mosquitoes involved and animal environment that may “protect” humans from being bitten as pointed out by Komar [91]. Other biases may modify the results of risk factors studies: horse cases are often detected by surveillance networks: results should be interpreted in the light of these network sensitivities. Moreover, horses living in endemic areas rarely show clinical cases: clinical cases reporting may not be representative of the WNV circulation, thus of the risk for humans to be infected. Indeed, from an epidemiological point of view, the incidental host infection pattern is a biased representation of the transmission cycle between wild birds and mosquitoes. Considering that host infection is the result of the interaction between a triptych “host-vector-virus” and its environment, a complete model, including all these parameters could explain the way transmission occur and help identifying the key factors that determine the spatio-temporal dynamic of transmission.

The second group of papers was based on the assumption that the geographic variations of WNV transmission risk result from those of abundance of competent birds and vectors. The geographic distribution of the disease also depends on human and horse spatial density. Modelling vector/bird abundance and human and horse spatial density, the final goal is to combine these geographical layers to compute an indicator for the local WNV transmission and WN disease risk. Regarding to mosquito abundance modelling, the above mentioned parameters linked to mosquito abundance (temperature, rainfall, NDVI), expert opinion and bird-baited trapping data were used to derive maps of the variations of mosquito abundance. Because of the strong link existing between mosquito biology and climate as well as the limited capacities of active flight of the European competent mosquito species, these models are accurate enough to correctly predict in time and space the geographic variations of mosquito abundance. Oppositely, no statistical model of the geographic variations of wild bird abundance was found, probably because (i) the collection of wild bird trapping data is very expensive, (ii) bird flight span is much more larger than for mosquito and (iii) individually speaking, habitat is varying with the bird life cycle. Furthermore, within a given species, the proportion of birds that migrate may vary according to the population, as well as the locations of the breeding sites. 

In several of the examined studies, maps of vector/bird abundance and of human/horse densities were combined to derived risk maps of WNV transmission or of WNF occurrence. The risk computation procedures were either based on a descriptive approach and expert opinion [44], or on a weighted linear combination of these [48]. An alternative methodology has recently been proposed allowing the mathematical combination of data and expert opinion to produce unbiased information for decision making. Multi Criteria Evaluation (MCE) or Multi Criteria Decision Analysis (MCDA) could be applied to WNF in the Mediterranean context [92,93]. Indeed this methodology allows creating maps in data-poor environments by incorporating all what is already known about disease transmission [93]. MCDA has successfully been used for the computation of risk maps for both directly transmitted and vector borne diseases, namely Avian Influenza in Southeast Asia [94] and Rift Valley fever in Italy [95].

The above landscape epidemiology studies compute risk indices based on the combination of bird and mosquito abundance indices. These risk indices are assumed proportional to the actual infection risk. However, if these studies investigate how the infection risk is shaped by the spatial co-occurrence of vectors and hosts, they do not explicitly take into account the temporal aspects of pathogen transmission (besides the computation of season-specific risk maps for example). Indeed, WNV transmission results from the interaction of the three main components of the epidemiological cycle, *i.e.*, virus, host and vectors. These components and their intrinsic characteristics (immune system, competence, virulence, feeding behavior…) are included in an environment (climatic, animal and human), that influence them. This environment also influences the intensity of the interactions between components, thus the transmission pattern. Beside the identification of the vectors and hosts, an accurate quantification of the contribution of different host species to viral amplification requires data on mosquito feeding patterns and host abundance from the same place and time, combined with information on the duration and intensity of host infectiousness [6]. This complexity may not be adequately summarized by a simple linear combination or a ratio. Among the examined papers, the complexity of WNV transmission has been addressed in a third group of studies, with 3 distinct approaches: studies dedicated to the “bird side”, that investigate the respective roles of wild bird species in WNV transmission, studies dedicated to the “vector side”, dedicated to the feeding behavior of mosquitoes, and process-based modeling studies that investigate WNV transmission dynamics.

*Culex* *modestus*, *pipiens* and *univittatus* are probably the main mosquito species involved in WNV transmission in the Mediterranean Basin. However several other species may be implicated [96]. Given the diversity of the bio-ecology of mosquitoes, the identification of the main vectors is a key component of surveillance and control actions in a given area. To infer on the implication of a given mosquito species in WN transmission, one should prove that this mosquito is laboratory competent, abundant enough during the transmission period and that its feeding behaviour mainly includes birds, human and/or horses. The third requirement is the virus isolation from field collected individuals [97]. Field capture surveys are also used to describe the population dynamic but the cost of these surveys does not allow replicating them as often as needed for the identification of periods and areas at risk. Beside laboratory experiments that may demonstrate the competency of a mosquito species and partially help analyzing the feeding behavior, the analysis of the relationship between mosquito abundance and environmental-climatic and landscape- covariates as well as the modeling of population dynamics are needed to predict in time and space the suitability of a given area for the occurrence a WN outbreak.

Regarding to the reservoir hosts, the top 15 most infectious hosts for WNV span 12 wild bird families in north America [98] and may be implicated in WN cycle. In the Mediterranean context however, knowledge is lacking and the identification of the species involved is another critical issue. Similarly to mosquitoes, a bird species must meet several requirements to be involved in WN cycle: to develop a sufficient viraemia to allow transmission to mosquitoes without dying, to be abundant enough, and to show a high mosquito feeding utilization index [99]. Estimates of reservoir competence may be obtained thanks to laboratory experimental infection assays for the most common wild bird species (e.g., in [100]). Such experiments have been conducted on few European species [101,102,103,104]. However, the cost of these experimental studies makes their generalization unrealistic, and modeling approaches are needed to estimate the reservoir competence of bird species from species-level covariates. The statistical analysis of species-level seroprevalence data according to species life history traits is a promising approach that could eventually lead to fully take into account the impact of bird species assembly on WNV circulation risk at a given place. However, the link between anti-WNV seroprevalence in a given bird species and reservoir competence of that species is not straightforward and laboratory studies are needed to quantify key parameters (such as the viraemia duration and intensity) in the most common European wild bird species 

Process-based models are the appropriate tools that could allow integrating the impacts of bird and vector biodiversity on WNV transmission dynamics and on the variations in time and space of the risk of WNV transmission in human and horse. Furthermore, 2 lineages are now incriminated, the interactions of which could alter WNV transmission dynamics. To date, the use of process-based models for the study of WNV transmission dynamics is quite limited in Europe, with a single, non-spatialized model, in which the representation of bird and vector biodiversity is strongly simplified [52]. Contrary to statistical models of which predictions are only reliable for conditions that fall within the observed ranges of parameters, process-based model are not linked to specific conditions and can thus be used for prediction. Further process-based modelling studies are thus needed to compute realistic predictions of the time and space variations of WNF risk. Besides compartmental models, other formalisms such as agent-based models [105] could be useful to fully integrate birds and mosquito biodiversity, as well as the behaviour of incidental hosts that determines their exposure.

## 5. Conclusions

WNF is an ecology-dependant disease. The way these above-mentioned factors combine and trigger outbreaks remain partially unknown. Building predictive models that link environmental variables and WNF risk is however essential to the understanding of how environmental changes, human-induced or not, will affect the dynamics of its transmission. Although land cover change has often been tied to spatial variation in disease occurrence, the underlying factors driving the correlations are complex, limiting the generalization of these results for disease prevention and control. Furthermore, temporal and dynamic aspects should be considered, since the success of the transmission process depends on the contact between host and vectors, thus, among other, on their population mobility and dynamic. A lot has been done, and landing is not so far away. However several elements of biological and ecological knowledge are still missing and experimental studies are needed to quantify key parameters. In this respect, it is important to emphasize the mutual input of biological/ecological knowledge and modeling approaches (Figure 1): modeling studies lead to the identification of key points and knowledge gaps that should be investigated through observational or experimental studies. In turn, biology provides the necessary pieces to build and validate models [106]. 

In conclusion, to properly reproduce WNV transmission dynamic at a given location and implement adapted surveillance systems or test the efficiency of control scenario such as insecticide spraying, there is a need to combine both spatial and dynamic approaches, integrating into a location-specific epidemiological cycle the available ecological knowledge of the WNV transmission processes: the dynamics of infection in mosquitoes and birds, the bird and vector population dynamics, and the time variations of exposure in incidental hosts.
